# What Can We Learn about Workplace Heat Stress Management from a Safety Regulator Complaints Database?

**DOI:** 10.3390/ijerph15030459

**Published:** 2018-03-06

**Authors:** Alana Hansen, Dino Pisaniello, Blesson Varghese, Shelley Rowett, Scott Hanson-Easey, Peng Bi, Monika Nitschke

**Affiliations:** 1 School of Public Health, The University of Adelaide, Adelaide, SA 5005, Australia; alana.hansen@adelaide.edu.au (A.H.); blesson.varghese@adelaide.edu.au (B.V.); scott.hanson-easey@adelaide.edu.au (S.H.-E.); peng.bi@adelaide.edu.au (P.B.); 2 SafeWork SA, Government of South Australia, 33 Richmond Road, Keswick, SA 5035, Australia; shelley.rowett@sa.gov.au; 3 Department for Health and Ageing, Government of South Australia, 11 Hindmarsh Square, Adelaide, SA 5000, Australia; monika.nitschke@sa.gov.au

**Keywords:** occupational health, heat exposure, qualitative

## Abstract

Heat exposure can be a health hazard for many Australian workers in both outdoor and indoor situations. With many heat-related incidents left unreported, it is often difficult to determine the underlying causal factors. This study aims to provide insights into perceptions of potentially unsafe or uncomfortably hot working conditions that can affect occupational health and safety using information provided by the public and workers to the safety regulator in South Australia (SafeWork SA). Details of complaints regarding heat exposure to the regulator’s “Help Centre” were assembled in a dataset and the textual data analysed thematically. The findings showed that the majority of calls relate to indoor work environments such as kitchens, factories, and warehouses. The main themes identified were work environment, health effects, and organisational issues. Impacts of hot working conditions ranged from discomfort to serious heat-related illnesses. Poor management practices and inflexibility of supervisors featured strongly amongst callers’ concerns. With temperatures predicted to increase and energy prices escalating, this timely study, using naturalistic data, highlights accounts of hot working conditions that can compromise workers’ health and safety and the need for suitable measures to prevent heat stress. These could include risk assessments to assess the likelihood of heat stress in workplaces where excessively hot conditions prevail.

## 1. Introduction

Each state and territory in Australia has a work health and safety regulator to oversee workplace practices, educate, enforce laws, and ensure compliance [1]. In the state of South Australia (SA), these responsibilities are under the jurisdiction of SafeWork SA (SWSA), which was established in 2005. At that time, the Occupational Health Safety and Welfare (SafeWork SA) Amendment Act was passed in state parliament, following many changes to the original South Australian Workers Rehabilitation and Compensation Scheme that commenced in 1987 [2].

The present role of SWSA is to provide work health and safety and industrial relations services to South Australian workplaces and reduce the incidence of injury through engagement and action, and the provision of information, assistance, education, and compliance activities to promote safety. Inspectors are responsible for ensuring that safety standards are met and that appropriate enforcement action is taken if breaches of the laws are detected; while advisors provide information, advice, and support to meet individual workplace needs [1]. One of the high-risk workplace issues recognised by SWSA is heat exposure.

The impacts of extreme heat on population health in Australia are well documented [3,4,5,6,7]. With hot summers, SA is no stranger to prolonged periods of hot weather that have resulted in reported increases in morbidity and mortality in recent years [4,8,9]. The spectrum of heat illnesses include relatively minor heat rashes to more serious heat exhaustion, which, if untreated, can lead to heat stroke with central nervous system involvement progressing to multi-organ dysfunction syndrome affecting major organs, including the brain, kidneys, and heart, and can be fatal [10].

Whereas studies have inferred high heat-susceptibility in the aged, frail, isolated, and ill in SA [8,11] those of working age are not risk-free. Indeed, during a severe 2009 heatwave in Adelaide, the state’s capital, there was a 37% increase in mortality for those in the 15–64 years age group, whereas no significant increase in older age groups was detected [4]. Work-related ambulance callouts have also been shown to increase in some Adelaide suburbs during heatwaves [12].

That extreme heat poses a risk to the health and safety of people in the SA workforce has been asserted by Xiang et al., who have shown an increase in occupational compensation claims during days, and extended spells, of high ambient temperature [13,14]. Often outdoor workers can be a group particularly at risk on hot days due to (a) high exposure to direct sunlight and heat, and (b) the physical and exertional nature of the work they can be undertaking. Combined, these factors can overwhelm the body’s thermoregulatory ability to maintain its core temperature within the normal range, with symptomatic consequences that range from thermal discomfort to the serious health outcomes previously mentioned. However, uncomfortably high heat-exposure is not restricted to outdoor workers. Many employees in indoor settings such as factories, kitchens, and foundries work in hot environments without air conditioning and are unable to take advantage of hot weather cease-work policies that can apply to their counterparts working in well-regulated outdoor industries.

Another less obvious but still concerning health and safety problem associated with working in hot environments is the propensity for heat-related occupational injuries. These can arise for reasons including fatigue, loss of concentration, dehydration, slippery hands, or impaired vision due to sweating, or tools being too hot to handle safely. Xiang et al. noted several types of heat-associated injuries during heatwaves including burns, lacerations, wounds, and amputations [13]. In another study based in Adelaide, the same authors noted an increase in daily injury claims with each 1 °C increase in maximum temperature up to 37.7 °C [14]. Findings were similar in a study based in Victoria where positive associations were found between temperature and acute work-related injuries in young workers, male workers, and people undertaking heavy physical work on hot days [15].

Although there are state and national guidelines for employers on thermal safety and the prevention of occupational heat stress in workers, there are no enforceable laws. Under the South Australian Work Health and Safety Act 2012, “A person conducting a business or undertaking at a workplace must ensure, so far as is reasonably practicable”, that “workers carrying out work in extremes of heat or cold are able to carry out work without risk to health and safety” [16]. In effect, persons can legally continue to conduct a business or undertaking in extreme conditions if they subjectively deem those conditions to pose no undue risk to health and safety.

“Dangerous” incidents including those causing death, serious injury, or illness, are “notifiable”, and national Work Health and Safety laws require they be reported to the regulator immediately. Penalties apply for non-compliance [17]. However, anecdotally a considerable number, if not most, incidents are not serious enough to be notifiable and many heat-related illnesses go unreported [18]. While these incidents may cause physical harm, illness, or distress, it is difficult to assign causal inference to heat exposure when details are not officially recorded.

Workers can feel powerless or reluctant to approach management regarding grievances about work conditions and safety cultures. However, concerned members of the public and workers in SA are able to voice their concerns to SWSA regarding conditions they consider to pose a threat to workplace health and safety. During 2015–2016, there were a total of 44,631 contacts made to SafeWork SA, 10,357 notifications, and 22 formal complaints that were subsequently resolved through the formal customer feedback process [19]. Details of telephone calls lodged with SWSA are compiled in a database. This information may provide valuable, yet unofficial, insights into heat risks and unsafe conditions in workplaces.

In this unique study, we examined SWSA data garnered from calls about hot work environments. The aim was to provide insights into potentially unsafe or uncomfortably hot working conditions and work practices. This ‘naturalistic data’ [20] has been collected without being affected by the actions of the researchers, and thus provides a novel insight into the lived experience of workers. These findings may be useful to business operators, policymakers, and health and safety officials charged with undertaking risk assessments and prevention measures for occupational heat stress.

## 2. Materials and Methods

In early 2006, SWSA established a Help and Early Intervention Centre (this later became the “Help Centre”), which members of the public could contact and be provided with advice on health and safety matters, or lodge a formal complaint. All official complaints and notifications to the regulatory safety agency that justified the creation of a record were kept; however, from 2009, a phone call logging system was put in place that allowed for a record of call details. Some calls are logged as enquiries and some are classified as official workplace complaints or notifications of injury or dangerous occurrence. SWSA offers callers an option for intervention for the latter. Notifications and complaints are assessed and triaged for response priority [19]. Action in relation to a workplace complaint varies from a phone call to statutory notices in line with the current compliance and enforcement policy. For example, if evidence was provided to the regulator regarding action already taken to address the subject of the complaint, the matter may just be logged and evidence filed. However, if the issue was triaged to an inspector, it would usually result in a visit to the site to verify the particulars of the complaint (or notifiable incident) and could have resulted in the issue of statutory notices to achieve compliance with OHSW/WHS laws.

Using the key word ‘heat’, the details of the lodged complaints and notifications from 2002 to 6 January 2017 were searched by an SWSA officer for those relating to concerns about thermal conditions. Excluded were approximately 450 “answered” calls where persons enquired about heat-related topics and, after speaking with SWSA, were satisfied that they could deal with the matter through their workplace risk management processes. For these calls—usually regarding cut-off temperatures to stop work or the number of breaks required in hot weather—only brief data is reported. All relevant records were extracted and compiled in a database, with identifying information of people or companies removed. The resultant dataset was sourced from SWSA.

The data were imported into NVivo (QSR International Pty Ltd., Doncaster, Australia), a software analysis tool to aid in the analysis of qualitative data. Using descriptive statistics, and thematic analysis augmented by a content analysis [21], the data were interrogated to search for tractable patterns in the text. Textual sections of data were coded accordingly into aptly named ‘nodes’, which were eventually refined into a final set of themes.

Ethics approval for the study was sought from the Human Research Ethics Committee of the University of Adelaide (Ethics Approval No. H-2016-085), which deemed there to be no ethical issues raised. This study forms part of a larger study with ethics approved by the Human Research Ethics Committees of The University of Adelaide, Queensland University of Technology, Monash University, and the University of Western Australia.

## 3. Results

**Figure 1:**
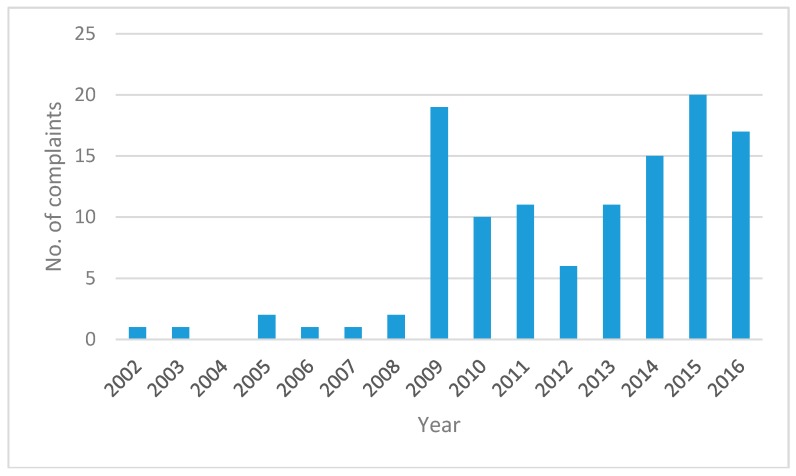
Recorded heat-related formal complaint calls to SafeWork SA (known as Workplace Services pre 2005) over the period 2002–December 2016. The Help Centre was established in 2009.

There were 118 records mentioning “heat” in the Help Centre dataset; eight in the period from November 2002 to March 2008, prior to the establishment of the “hotline”. Most (37) calls were logged in the month of January, 21 in December, 20 in February, 14 in November, and 12 in March. The remaining 14 calls were logged in the cooler months (April to October). An extreme unprecedented heatwave occurred in early 2009 [22] coinciding with 9 calls from January to March of that year. Since the establishment of the Help Centre in 2009, an apparent increasing trend in heat-related calls received to the end of 2016 has been noted, as shown in Figure 1.

The data in the records were often of poor quality with details too scant to analyse—one entry comprised only two words, whereas the most comprehensive contained 506 words. A simple content analysis revealed the top 50 most frequent words of at least three letters in the dataset were, ‘heat’, “workers”, and “working” (data not shown). Thematic analysis of the data revealed three main themes: work environment, health effects, and organisational issues.

### 3.1. Work Environment

Of the total 118 calls, more than half (63) related to indoor work, 38 to outdoor work, and 17 were non-classifiable due to limited information provided. The indoor workplaces included stores, kitchens, sheds, restaurants, factories, vehicles, workshops, a roadhouse, a glasshouse, and a warehouse. For some, the nature of the workplace was not stated, but details alluded to an indoor environment. Air conditioning (inadequate or non-existent) was a major subtheme of these calls. Some complainants rang with concerns that air conditioning was “not working properly”, had “broken down some time ago and has not been fixed”, or that evaporative cooling was ineffective. Another caller said that a once-working air conditioner had been removed for economic reasons. Additionally, some workplaces reportedly had “industrial fans”, some had “no ventilation”, and others had “no air conditioning or heating”.

Some callers cited (unverified) indoor temperatures (in degrees Celsius), both estimated and measured. Examples are as follows:

The air conditioner in the workshop has been tested with a thermometer in the vent and is alleging that the air blowing out is at 52 °C.

(#1)

The thermometer in the shed was reading 55 °C.

(#26)

The kitchen thermostat is reading at between 60–70 °C.

(#116)

Comparatively fewer calls about outdoor work environments were received. One complainant worked outdoors “even though the temperature was in the high 30’s” and another “in the sun in 42 °C heat”. The main subtheme for outdoor environments related to the lack of sun protection such as hats, and no provision to seek shade or “get out of the heat”.

### 3.2. Health Effects

Callers to the Help Centre cited a range of actual health effects and symptoms attributed to hot work environments. Some were merely raising concerns about potential health effects in workers or members of the public in current or upcoming situations—e.g., a scheduled sporting event in extreme heat, or young European fundraisers door knocking “in the searing heat of the Adelaide sun”. The health consequences mentioned by callers ranged from minor (e.g., pale, feeling tired, and distressed) to headaches, fainting, muscle fatigue, and exhaustion. Other, more serious cases were cited with individuals being dehydrated, vomiting, or losing consciousness. Some workers required transport in ambulances and visits to general practitioners or hospitals. There was also a report of a worker who suffered chest pains while working in extreme heat, was transported by ambulance to seek health care, returned to work, and later died.

The term ‘heat stroke’ was used by some callers when something more minor on the heat-related illness continuum may have been intended. For example,

The next day when he returned to work he was suffering ill effects from heat stroke from the day before.

(#108)

…suffered heat stroke a week before Xmas, and thinks she may be suffering from it again.

(#12)

Nevertheless, actual cases of heat stroke were mentioned:

…worker was admitted to hospital with extreme heat exhaustion (heatstroke).

(#46)

### 3.3. Organisational Issues

Several issues were mentioned by complainants that related to organisational issues and management. In the majority of these, callers reported that, despite the hot conditions, workers were “required to work long hours in the heat” and requests for extra breaks were denied. In one case, there was a bonus incentive to keep working and in another there were threats of job losses for workers requesting breaks.

…the foreman’s attitude was that, if he stops work, don’t bother coming back the next day.

(#10)

Some callers told of supervisors whose attitudes were seemingly unreasonable, as in the case of a worker in a rural area who had lost consciousness twice in the heat. The supervisor refused to let the worker be taken to an air-conditioned room and was then reluctant to assist in helping transport them to an awaiting ambulance some distance away. In other cases of poor management practices, a health and safety representative was temporarily suspended for filing a report, some workers were “forced to work installing insulation in house roofs in extreme heat”, and on one occasion management was “pushing people to work past their capacity”. Furthermore, some callers reported that on some worksites there was no “drinking water for employees” or that the water was “too hot to drink on hot days”. In some instances, workers reportedly had to supply or buy their own water.

The safety culture of workplaces came into question in several calls to the Help Centre. Personal protective equipment (PPE), safety training, first aid, and hot weather policies were among the issues raised. Two callers complained about having to wear full PPE, but more said that PPE “to protect against the heat” was not provided by the employer. One frustrated worker in the transport industry clearly stated his/her views on employers’ responsibility for their workers’ health and safety:

…time for drivers being responsible for their safety and their own PPE are done … it should be the companies [sic] responsibility to ensure my safety whilst on site without being put in heat/cold conditions that cause sickness … it’s unfair and wrong.

(#15)

There were also a small number of calls relating to a lack of first aid facilities or provider, and no or minimal, safety training. Several complainants raised the issue of no hot weather policies in place or that policies were not adhered to. It is particularly concerning that an insulation installation company reportedly had no hot weather policy. Views of policies were unclear, however, as one caller claimed that, although there was no heat policy, the workers could “start earlier and knock off earlier”. Acknowledgements of other heat stress mitigation measures included workers being provided with “ice packs and wet towels to keep cool”, powdered electrolytes, and “plenty of cold water”.

## 4. Discussion

This study summarises the calls to a safety regulator from members of the public with concerns about occupational heat exposure. It highlights some alarming and potentially harmful thermal environments in which employees work, and questionable safety cultures in some workplaces. Notably, there was an increasing trend in the number of these calls over the study period. However, it cannot be determined how much of this increase can be attributed to increasing knowledge of the existence of the Help Centre facility over this period.

The majority of calls related to indoor work environments. This is in contrast to a considerable body of literature identifying outdoor workers amongst those vulnerable during extreme heat [18,23,24]. Whereas some complaints in this study related purely to conditions causing thermal discomfort, this is not covered under the South Australian Work Health and Safety Act 2012, which specifies “extremes of heat or cold” [16]. These terms could of course be subjective. Nevertheless, clearly some indoor workers in this study were working in extremes of heat if temperatures were as high as reported. Hot indoor environments can occur where there are heat-generating sources and/or it is neither practical nor economically viable for management to install and operate cooling devices during periods of extreme heat. Air-conditioned businesses will likely be faced with soaring electricity bills, as SA reportedly has the highest power prices in the world [25]. By economising on energy expenditure and minimising air conditioner usage, employers could potentially jeopardise workers’ health, safety, and welfare during extreme heat. It is therefore important that policies consider the thermal safety of all workers, not just those exposed to solar radiation. In some instances, industrial fans may be an option although in this study there were reports of some workplaces with no ventilation at all. This can impede workers’ ability to lose body heat gained from the environment, as low air velocity impairs the evaporation of sweat, the major heat loss mechanism when ambient air temperature exceeds skin temperature [26].

Under the South Australian Work Health and Safety Act 2012, employers need to ensure “so far as is reasonably practicable” that the thermal environment is such that workers “are able to carry out work without risk to health and safety” [16]. The issues raised by callers highlight that, despite educational material being available and regulations existing for persons conducting a business or undertaking, these can be ignored or not understood, posing conditions that can jeopardise the health and safety of workers in harsh thermal environments. In this study, some workers reportedly became ill, and in one case there was a death.

Several studies across Australia [13,14,15,23,27] have shown that high temperatures can compromise the welfare of workers, potentially resulting in injuries, heat illnesses, or death. However, our findings show there appears to be a lack of understanding amongst callers about the spectrum of heat-related illnesses and the severity of heat stroke. This information should be included in health and safety inductions for those likely to encounter hot working conditions. Heat illnesses are largely preventable [28] yet several barriers to successful occupational heat-health and safety measures exist. These include both a lack of understanding of the risks associated with heat exposure and the attitude that during summers in Australia heat is a hazard that cannot be avoided [29]. Furthermore, in many at-risk industries that are male-dominated concerns for one’s safety may be viewed as a sign of weakness [29,30] with peer pressure to “toughen up”.

Mitigation and prevention strategies need to be available for those in occupations where excessively hot conditions prevail. Cool drinking water must be on hand, suitable PPE provided, a shaded area if possible, adequate and regular work breaks allowed, and training for supervisors and employees on how to recognise and respond to heat-related illnesses [28]. Heat policies may need to be enforced in extreme conditions. Callers highlighted a failure of some managements to provide or implement these actions, which may imply discordant views about who is ultimately responsible for a worker’s health and safety. Lao et al. (2016) found that workers coped better with heat exposure risks if those in management were flexible toward heat stress prevention and allowed workers to self-pace. Indeed, without personal experience, management may underestimate the level of heat exposure experienced by staff. Although slowing down and taking extra breaks can result in a loss in productivity and work capacity, workers’ health and safety should take priority on the occasions when conditions are extreme [29,30].

It is likely that most health and safety representatives are well aware of the risks of heat exposure on occupational health, yet heat stress continues to occur in workplaces, as a result perhaps of inadequacies in safety training to alert workers to heat as an environmental hazard, or a perceived need to keep working while ignoring physiological warning signs. Demographic issues such as an ageing workforce combined with an increase in individual and consecutive hot days may also be contributing factors. Another possibility is that compliance with organisational policies may diminish the autonomous or self-determined motivation of workers to reduce the risk of heat stress, consistent with the self-determination theory [31]; therefore ways should be found to support autonomous motivation for preventive behaviours.

Risk assessments can be undertaken by occupational hygienists or other qualified personnel to assess workplace conditions, the work being undertaken, and the likelihood of heat stress occurring. Workers, and health and safety representatives, need to be part of the consultation process when risk assessments are undertaken. Air temperature alone may not be a suitable indicator of heat risk as several factors may need to be considered [28]. While complex metrics such as the Thermal Work Limit [32,33] can be calculated, heat stress indices such as Wet Bulb Globe Temperature that take into account air temperature, humidity air movement, and radiant heat [34] can more easily be measured with instruments, calculations or mobile phone applications. The findings of this study clearly show the importance of thermal safety for all workers in hot conditions not just those exposed to solar radiation. Risk assessments should therefore also be available for hot indoor environments that need adequate air flow and access to drinking water, and an allowance for workers to self-pace as required.

The limitations of this study are acknowledged, particularly with regard to the data quality and quantity. The data comprised telephone operators’ brief notes outlining callers’ concerns, so there may be errors in, or misinterpretations of, the data. It should also be noted that callers’ claims about thermal conditions were unverified at the time of the call. Official notifications or complaints however, would have been followed up by the regulator. We cannot make assumptions about how widespread the problem is, as these heat-related calls only represented a small proportion of the total calls received by the regulator. Additionally, as the system was not specifically designed for reporting heat complaints, there would likely have been many unreported cases of occupational heat stress. Lastly, as a result of the search methods used, some trivial and uninformative records were included in the final dataset. Nevertheless, the data contained valuable “naturalistic” accounts and public perceptions of occupational heat risks unlikely to have been aired in any other forum and previously noted only by health and safety managers. The value of this type of data as a source of intelligence gathering on health and safety risks has been demonstrated.

## 5. Conclusions

This unique study, accessing data from a safety regulator complaints database, provides an insight into the public’s and workers’ accounts of hot work environments where health and safety may, and in some cases was, compromised. Reports of thermal conditions perceived to be potentially unsafe to health, and poor management practices/organisational issues, featured strongly amongst callers’ concerns. This is despite regulations in place and a plethora of guidance material on heat risks available for persons conducting a business or undertaking. Hot indoor work environments featured strongly in the complaints possibly due to the high cost of operating cooling systems. For outdoor workers the provision of PPE for protection from the sun and heat was mentioned. Clearly, to mitigate the effects of heat, workers need to be able to autonomously undertake protective behaviours without fear of reprisals, and employers need to be aware of their responsibilities. This is particularly important in an era of rising ambient temperatures [35] and increasing energy prices, both of which may impact on thermal conditions and occupational health and safety in work environments.
